# Prediction of the impact of anxiety on atrial fibrillation recurrence after radiofrequency catheter ablation based on heart rate variability

**DOI:** 10.3389/fsurg.2025.1653123

**Published:** 2025-09-23

**Authors:** Yufei Ren, Hua Zhang, Yingji Tian

**Affiliations:** ^1^ Department of Internal Medicine, Beijing Huilongguan Hospital, Beijing, China; ^2^ Department of Medical Imaging Center, Beijing Huilongguan Hospital, Beijing, China; ^3^ Department of General Practice, Beijing Changping District Huilongguan Community Health Service Center, Beijing, China

**Keywords:** atrial fibrillation (AF), radiofrequency catheter ablation (RFCA), recurrence, heart rate variability (HRV), anxiety

## Abstract

**Background:**

Radiofrequency catheter ablation (RFCA) can significantly improve the prognosis of patients with atrial fibrillation (AF); however, the postoperative recurrence rate remains high. Therefore, identifying accurate predictors of recurrence after RFCA holds important clinical value.

**Methods:**

This retrospective study included 180 patients with AF who underwent RFCA. Patients were grouped by one-year recurrence status. Univariate analysis was conducted to compare demographic and clinical characteristics between the two groups. Cox proportional hazards models and Kaplan–Meier survival curves were used to assess the impact of heart rate variability (HRV), anxiety, and their interaction on recurrence. Predictive performance was evaluated with receiver operating characteristic (ROC) curves. Stratified analyses were performed to explore whether the effect of anxiety on recurrence varied by HRV levels.

**Results:**

Compared with the non-recurrence group, the recurrence group had higher prevalence of persistent AF and heart failure, longer AF duration, and more severe left atrial structural burden (i.e., higher EFT, LAD, and LAVI values). Multivariate Cox analysis identified that both HRV and anxiety were independent risk factors for recurrence, and their interaction term also had significant predictive value (HR > 1, *P* < 0.05). Kaplan–Meier analysis indicated that patients with low HRV and high anxiety had the lowest recurrence-free survival rate. ROC curve analysis revealed that the combined HRV-anxiety interaction model yielded an AUC of 0.745, indicating a certain predictive advantage over individual indicators. Stratified analysis further confirmed that the recurrence risk associated with high anxiety was more pronounced in the low HRV group.

**Conclusion:**

HRV and anxiety were identified as independent predictors of AF recurrence following RFCA, with a significant synergistic interaction observed between the two. Their combined assessment may enhance the accuracy of recurrence risk prediction and provide a foundation for the development of individualized intervention strategies.

## 1 Introduction

Atrial fibrillation (AF) is a common arrhythmia characterized by rapid and irregular electrical activity in the atria (1, 2). It is associated with increased risks of stroke, heart failure, and all-cause mortality, and seriously affects patients' quality of life (3–6). Its pathogenesis involves changes in cardiac electrophysiology, structural remodeling, and neuroendocrine regulation (7). Current treatment includes drug therapy, such as anticoagulants to prevent stroke and antiarrhythmic drugs to restore and maintain sinus rhythm, and non-drug therapy, including interventional methods such as electrical cardioversion, maze surgery, left atrial appendage occlusion, and radiofrequency catheter ablation (RFCA) (8, 9). Despite these treatments, AF recurrence remains common, highlighting the need to further clarify risk factors to guide precise prevention and intervention.

RFCA is an important and guideline-recommended treatment for atrial fibrillation (AF) (10). According to the 2024 ESC and 2023 ACC/AHA guidelines (11, 12), RFCA is indicated for patients with symptomatic paroxysmal or persistent AF who have failed or are intolerant to antiarrhythmic drugs, and it is increasingly considered as a first-line option in selected cases. However, the recurrence rate after radiofrequency ablation remains significant. Studies have shown that the incidence of postoperative recurrence ranges from 30% to 50% (13, 14). Therefore, in-depth exploration of the mechanisms influencing recurrence after RFCA has important clinical value for optimizing treatment strategies and improving patient prognosis.

Most existing studies focus on the independent predictive value of HRV (e.g., the association between reduced LF/HF ratio and recurrence) (15, 16), but the interaction between anxiety disorders and HRV and their synergistic impact on recurrence remains unclear. This study is the first to integrate a psychological factor (anxiety) with a physiological indicator (HRV) to explore their combined predictive value for recurrence, providing multidimensional targets for postoperative management and intervention.

## 2 Materials and methods

### 2.1 Study population

This retrospective study included patients with atrial fibrillation (AF) who underwent their first radiofrequency ablation (RFCA) between June 2022 and June 2024. Inclusion criteria were: (1) age over 18 years; (2) postoperative follow-up of ≥12 months with clear documentation of recurrence; and (3) first-time RFCA treatment. Exclusion criteria included: (1) Severe hepatic dysfunction (Child-Pugh Class C) or renal insufficiency (eGFR < 30 ml/min/1.73 m^2^); (2) Presence of severe systemic diseases, including malignancies, end-stage heart failure (NYHA Class III–IV), severe pulmonary dysfunction (GOLD Stage III–IV), systemic lupus erythematosus, or hematological disorders (e.g., aplastic anemia, leukemia); (3) Diagnosed severe psychiatric disorders (such as schizophrenia, bipolar disorder, or major depressive disorder) confirmed by a psychiatrist, or poor treatment compliance; and (4) missing key data.

Poor compliance was assessed based on documented history of non-adherence to medical instructions, irregular follow-up visits, refusal to undergo recommended examinations, or the investigator's judgment—after communicating with the patient—that they were unlikely to complete the treatment and follow-up as required by the study protocol.

### 2.2 Procedure

All patients underwent radiofrequency ablation with the aim of achieving pulmonary vein isolation (PVI), guided by a three-dimensional electroanatomical mapping system (CARTO® 3, Biosense Webster, USA). Based on intraoperative electrophysiological findings, additional ablation lines—such as the left atrial roof line, mitral isthmus line, and tricuspid isthmus line—were applied when necessary to enhance efficacy. If sinus rhythm was not restored intraoperatively, external electrical cardioversion was performed before continuing the ablation until complete electrical isolation of both pulmonary veins was achieved. The procedural endpoint was the restoration of sinus rhythm and the confirmation of bidirectional conduction block at the pulmonary vein antrum, left atrial roof, mitral isthmus, and tricuspid isthmus. After ruling out contraindications, all patients received standard postoperative care including a 1-month course of a proton pump inhibitor (e.g., rabeprazole) to prevent gastrointestinal discomfort and mitigate esophageal injury risk, 3 months of oral anticoagulation therapy with rivaroxaban or edoxaban to prevent thromboembolism, and 3 months of oral amiodarone or dronedarone to maintain sinus rhythm and reduce early recurrence.

### 2.3 Data collection

Collected variables included age, gender, body mass index (BMI), presence of hypertension, diabetes, coronary artery disease, and heart failure; type of atrial fibrillation (paroxysmal or persistent); duration of AF and whether it was a first diagnosis; preoperative echocardiographic parameters including left ventricular ejection fraction (LVEF), epicardial fat thickness (EFT), left atrial diameter (LAD), and left atrial volume index (LAVI); and procedural parameters including ablation time, total procedure duration, total energy, and number of ablation targets. Based on transthoracic echocardiography and in accordance with current ESC and ACC/AHA/HFSA guidelines (17), heart failure was classified as HFrEF (LVEF < 40%) and HFpEF (LVEF ≥ 50%).

The GAD-7 questionnaire was completed during the routine preoperative evaluation 1–3 days before catheter ablation, and all patients were in a clinically stable condition at the time of completion. HRV data were obtained through 24-hour Holter monitoring. All patients were instructed to remain at rest during the recording, ensuring they stayed calm and relaxed to minimize external factors that could interfere with the HRV data. During data processing, automated software (Kubios HRV) was used for quality control of the raw data. Obvious artifacts, such as abnormal waveforms and noise in the ECG signal, were detected and removed to ensure data quality. Segments with irregular heartbeats or missing data were appropriately interpolated or excluded to maintain data continuity and validity. HRV analysis was primarily based on time-domain and frequency-domain methods. Time-domain indices included Standard Deviation of Normal to Normal Intervals (SDNN) and Root Mean Square of Successive Differences (RMSSD). The calculation method for SDNN is as follows: the mean value of all RR intervals is first calculated, then the difference between each RR interval and the mean value is computed, followed by averaging the squared differences, and finally taking the square root. The calculation for RMSSD is as follows: the difference between successive RR intervals is first calculated, then the squared differences are averaged, and the square root of the average is taken. In frequency-domain analysis, power spectral density estimation was performed using Fast Fourier Transform (FFT) to decompose the frequency and determine the power of low-frequency (LF) and high-frequency (HF) components. During the 12-month follow-up period after the procedure, all patients underwent a 12-lead electrocardiogram (ECG) and 24-hour Holter monitoring every month. Additional ECGs were performed if patients experienced symptoms such as palpitations or fatigue. Recurrence was defined as atrial tachycardia, atrial flutter, or atrial fibrillation lasting ≥30 s detected during the follow-up period.

### 2.4 Statistical analysis

All statistical analyses were performed using R version 4.4.1. Continuous variables were presented as medians (min–max) and compared using the independent t-test or Mann–Whitney U test. Categorical variables were expressed as frequencies (percentages) and compared using Fisher's exact test or the chi-square test. Patients were divided into high-anxiety and low-anxiety groups based on the median GAD-7 score, and into high-HRV and low-HRV groups based on the median LF/HF ratio. High anxiety and low HRV were coded as 1, while low anxiety and high HRV were coded as 0. Three Cox proportional hazards models were constructed: Model 1 included only anxiety, HRV, and their interaction; Model 2 further adjusted for age, gender, and BMI; Model 3 additionally adjusted for heart failure, AF type, duration, preoperative EFT, LAD, and LAVI. The outcome variable in all models was recurrence. Kaplan–Meier survival curves were used to estimate recurrence-free survival based on HRV level, anxiety level, and their interaction groups, with comparisons made using the log-rank test. Receiver operating characteristic (ROC) curves were used to evaluate the predictive ability of HRV, anxiety, and their interaction for recurrence. Stratified analyses were conducted to compare anxiety levels between recurrence and non-recurrence groups within high and low HRV strata, to assess the moderating effect of anxiety at different HRV levels. A two-sided *P*-value < 0.05 was considered statistically significant.

## 3 Results

### 3.1 Baseline characteristics of AF patients with and without recurrence

A total of 180 patients with atrial fibrillation (AF) were included in this study, with a median age of 63 years (range: 49–78). Among them, 122 were male (67.78%) and 58 were female (32.22%). The median body mass index (BMI) was 24.7 kg/m^2^ (range: 19.7–30.2). Comorbidities included hypertension in 80 patients (44.44%), diabetes mellitus in 30 patients (16.67%), coronary artery disease in 30 patients (16.67%), and heart failure in 19 patients (10.56%). Regarding AF type, 119 patients (66.11%) had paroxysmal AF, and 61 (33.89%) had persistent AF. The median duration of AF was 2.6 years (range: 0.2–5.5), and 101 patients (56.11%) were first diagnosed with AF. Echocardiographic parameters showed a median left ventricular ejection fraction (LVEF) of 53.8% (range: 48.3–59.5%), epicardial fat thickness (EFT) of 7.1 mm (range: 5.1–9.5), left atrial diameter (LAD) of 42.3 mm (range: 38.5–46.1), and left atrial volume index (LAVI) of 36.6 ml/m^2^ (range: 30.9–42.5). Procedural parameters included a median ablation time of 60 min (range: 38–75), total procedure duration of 136 min (range: 107–160), total energy of 20,917 joules (range: 12,070–28,621), and a median of 5 ablation targets (range: 2–9). The median RMSSD was 28.8 ms (range: 23.2–35.6 ms), the median SDNN was 47.2 ms (range: 35.9–57.9 ms), the median LF/HF ratio was 1.2 (range: 0.8–1.7), and the median GAD-7 score was 8 (range: 3–14). There were no statistically significant differences between the recurrence and non-recurrence groups in most demographic and common comorbidities, including age, sex distribution, BMI, and hypertension. However, the proportion of persistent AF was significantly higher in the recurrence group (53.33% vs. 20.00%, *P* < 0.001), as was the prevalence of heart failure (21.33% vs. 2.86%, *P* < 0.001). Additionally, the recurrence group had a longer AF duration (median 3.0 vs. 2.4 years, *P* = 0.0404). Structural burden of the left atrium was heavier in the recurrence group, with significantly higher EFT (7.7 mm vs. 6.9 mm, *P* = 0.00537), LAD (43.1 mm vs. 41.9 mm, *P* = 0.0215), and LAVI (37.8 ml/m^2^ vs. 36.1 ml/m^2^, *P* = 0.0191) compared to the non-recurrence group. The recurrence group showed significantly higher RMSSD compared to the non-recurrence group (29.4 ms vs. 28.5 ms, *P* = 0.0366), significantly lower LF/HF ratio (1.1 vs. 1.3, *P* = 0.0114), and significantly higher GAD-7 scores (10 vs. 8, *P* = 0.00537) (Table 1).

**Table 1:** Comparison of baseline characteristics between AF patients with recurrence and those without.

Variables	All patients (*n* = 180)	Non-recurrence (*n* = 105)	Recurrence (*n* = 75)	*P*-value
Age	63 (49–78)	64 (49–78)	61 (49–78)	0.278
Gender				0.5897588
Male	122 (67.78%)	69 (65.71%)	53 (70.67%)	
Female	58 (32.22%)	36 (34.29%)	22 (29.33%)	
BMI	24.7 (19.7–30.2)	24.8 (19.7–30.2)	24.4 (19.7–30.1)	0.727
Hypertension				0.388656
Yes	80 (44.44%)	50 (47.62%)	30 (40%)	
No	100 (55.56%)	55 (52.38%)	45 (60%)	
Diabetes mellitus				0.2235958
Yes	30 (16.67%)	14 (13.33%)	16 (21.33%)	
No	150 (83.33%)	91 (86.67%)	59 (78.67%)	
Coronary artery disease				0.2235958
Yes	30 (16.67%)	14 (13.33%)	16 (21.33%)	
No	150 (83.33%)	91 (86.67%)	59 (78.67%)	
Heart failure				0.000190535
Yes	19 (10.56%)	3 (2.86%)	16 (21.33%)	
No	161 (89.44%)	102 (97.14%)	59 (78.67%)	
Type of atrial fibrillation				6.85E−06
Paroxysmal	119 (66.11%)	84 (80%)	35 (46.67%)	
Persistent	61 (33.89%)	21 (20%)	40 (53.33%)	
Duration of atrial fibrillation	2.6 (0.2–5.5)	2.4 (0.2–5.5)	3.0 (0.4–5.4)	0.0404
First-diagnosed				0.4615779
Yes	101 (56.11%)	56 (53.33%)	45 (60%)	
No	79 (43.89%)	49 (46.67%)	30 (40%)	
Left ventricular ejection fraction, LVEF (%)	53.8 (48.3–59.5)	53.5 (48.3–59.5)	54.4 (48.5–59.4)	0.104
Epicardial fat thickness, EFT (mm)	7.1 (5.1–9.5)	6.9 (5.1–9.4)	7.7 (5.1–9.5)	0.00537
Left atrial diameter, LAD (mm)	42.3 (38.5–46.1)	41.9 (38.5–46.0)	43.1 (38.5–46.1)	0.0215
Left atrial volume Index, LAVI (ml/m^2^)	36.6 (30.9–42.5)	36.1 (30.9–42.5)	37.8 (30.9–42.4)	0.0191
Ablation time (min)	60 (38–75)	61 (39–75)	59 (38–75)	0.526
Procedure duration (min)	136 (107–160)	136 (107–160)	134 (108–160)	0.941
Total energy (J)	20,917 (12,070–28,621)	21,585 (12,323–28,621)	19,472 (12,070–28,390)	0.203
Number of ablation targets	5 (2–9)	5 (2–9)	6 (2–9)	0.101
Root-mean square successive differences, RMSSD (ms)	28.8 (23.2–35.6)	28.5 (23.2–35.6)	29.4 (23.3–35.6)	0.0366
Standard deviation of normal to normal intervals, SDNN (ms)	47.2 (35.9–57.9)	47.8 (36.2–57.9)	46.4 (35.9–57.9)	0.658
Low frequency to high frequency ratio, LF/HF	1.2 (0.8–1.7)	1.3 (0.8–1.7)	1.1 (0.8–1.7)	0.0114
GAD-7	8 (3–14)	8 (3–14)	10 (3–14)	0.00537

### 3.2 Cox proportional hazards models to analyze the interaction between HRV and anxiety on recurrence

In Model 1, which included only anxiety, HRV, and their interaction term, both anxiety (HR = 6.07, 95% CI: 2.05–17.94, *P* = 0.001) and HRV (HR = 7.98, 95% CI: 2.74–23.28, *P* < 0.001) were significant risk factors for recurrence, and their interaction term was also statistically significant (HR = 2.24, 95% CI: 1.20–4.19, *P* = 0.012), suggesting that anxiety modulates the association between HRV and recurrence. In Model 2, after adjusting for age, sex, and BMI, anxiety (HR = 6.31, 95% CI: 2.13–18.70, *P* = 0.001) and HRV (HR = 8.18, 95% CI: 2.80–23.90, *P* < 0.001) remained significant, and the interaction term (HR = 1.237, 95% CI: 1.01–1.51, *P* = 0.039) was still statistically significant, indicating that the synergistic effect between HRV and anxiety is independent of basic demographic factors. In the final Model 3, we included clinically relevant covariates with stronger statistical significance (including heart failure, type of atrial fibrillation, and EFT). The results showed that anxiety (HR = 5.812, CI: 1.96–17.22, *P* = 0.001) and heart rate variability (HRV) (HR = 6.887, 95% CI: 2.36–20.12, *P* < 0.001) remained independent predictors. Heart failure (HR = 2.449, 95% CI: 1.38–4.35, *P* = 0.002) and persistent atrial fibrillation (HR = 2.763, 95% CI: 1.73–4.41, *P* < 0.001) significantly increased the risk of recurrence. The interaction term between HRV and anxiety (HR = 1.190, 95% CI: 1.01–1.40, *P* = 0.038) also remained statistically significant, further supporting the joint predictive value of HRV and anxiety on recurrence (Table 2). Considering that dichotomizing continuous variables may lead to information loss, we conducted an interaction analysis using GAD-7 and LF/HF as continuous variables. Given that GAD-7 was positively associated with recurrence risk while LF/HF was negatively associated, we used - (LF/HF) to align the direction of associations. Results showed that GAD-7 scores (HR = 2.917, 95% CI: 1.669–5.100, *P* < 0.001) were significantly associated with recurrence, suggesting that higher anxiety levels increase recurrence risk. Increased - (LF/HF) values were also associated with higher recurrence risk (HR = 2.072, 95% CI: 1.070–4.011, *P* = 0.031). The interaction between GAD-7 and - (LF/HF) was significant (HR = 2.180, 95% CI: 1.094–4.346, *P* = 0.027), indicating a synergistic effect between the two. Specifically, when both high anxiety levels and low LF/HF values were present, the risk of recurrence increased markedly (Supplementary Table S1).

**Table 2:** The effect of the interaction between heart rate variability (HRV) and anxiety on recurrence was analyzed using a Cox proportional hazards model.

Term	HR	Std error	Statistic	*P* value	CI-Lower	CI-Upper
Anxiety	6.068	0.553	3.261	0.001	2.053	17.938
HRV	7.983	0.546	3.806	0.000	2.738	23.277
Anxiety*HRV	2.240	0.320	2.520	0.012	1.196	4.194
Anxiety	6.313	0.554	3.323	0.001	2.131	18.699
HRV	8.180	0.547	3.841	0.000	2.800	23.898
Age	0.983	0.014	−1.260	0.208	0.956	1.010
Gender	1.122	0.256	0.449	0.653	0.679	1.853
BMI	0.976	0.035	−0.682	0.495	0.911	1.045
Anxiety*HRV	1.237	0.103	2.065	0.039	1.011	1.514
Anxiety	5.812	0.554	3.179	0.001	1.962	17.215
HRV	6.887	0.547	3.530	0.000	2.357	20.121
HF	2.449	0.293	3.053	0.002	1.379	4.349
Type of atrial Fibrillation	2.763	0.238	4.262	0.000	1.733	4.405
EFT	1.193	0.101	1.738	0.082	0.979	1.454
Anxiety*HRV	1.190	0.084	2.071	0.038	1.009	1.403

### 3.3 The impact of anxiety, HRV, and their interaction on recurrence-free survival

**Figure 1:**
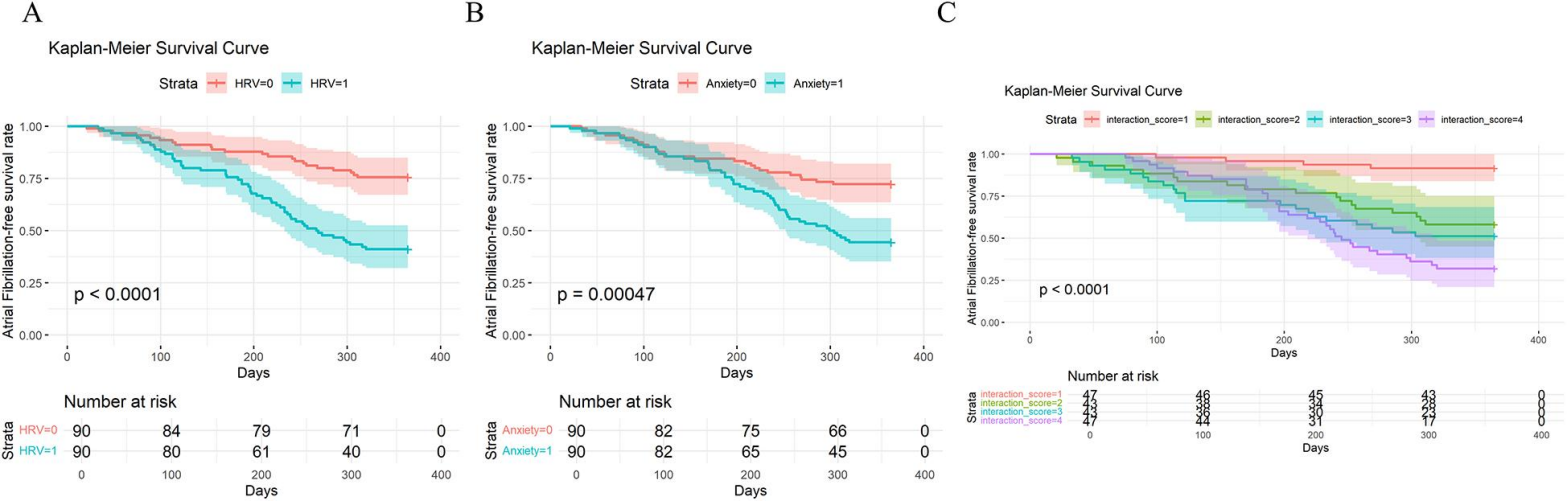
Kaplan–Meier analysis of recurrence-free survival in patients with atrial fibrillation (AF): **(A)** based on HRV levels (low HRV = 1, high HRV = 0); **(B)** based on anxiety levels (high anxiety = 1, low anxiety = 0); **(C)** based on the combined stratification of HRV and anxiety (0 = low anxiety & high HRV, 1 = high anxiety & high HRV, 3 = low anxiety & low HRV, 4 = high anxiety & low HRV).

Kaplan–Meier curves showed that the recurrence-free survival rate in the low HRV group was significantly lower than that in the high HRV group (Figure 1A). Similarly, patients in the high anxiety group had a significantly lower recurrence-free survival rate compared to those in the low anxiety group (Figure 1B). Notably, the group with both low HRV and high anxiety had the lowest recurrence-free survival rate, even compared to groups with only one abnormal factor. This further suggests a synergistic or interactive effect between HRV and anxiety (Figure 1C).

### 3.4 ROC curve analysis of the predictive ability of HRV, anxiety, and their interaction for AF recurrence

**Figure 2:**
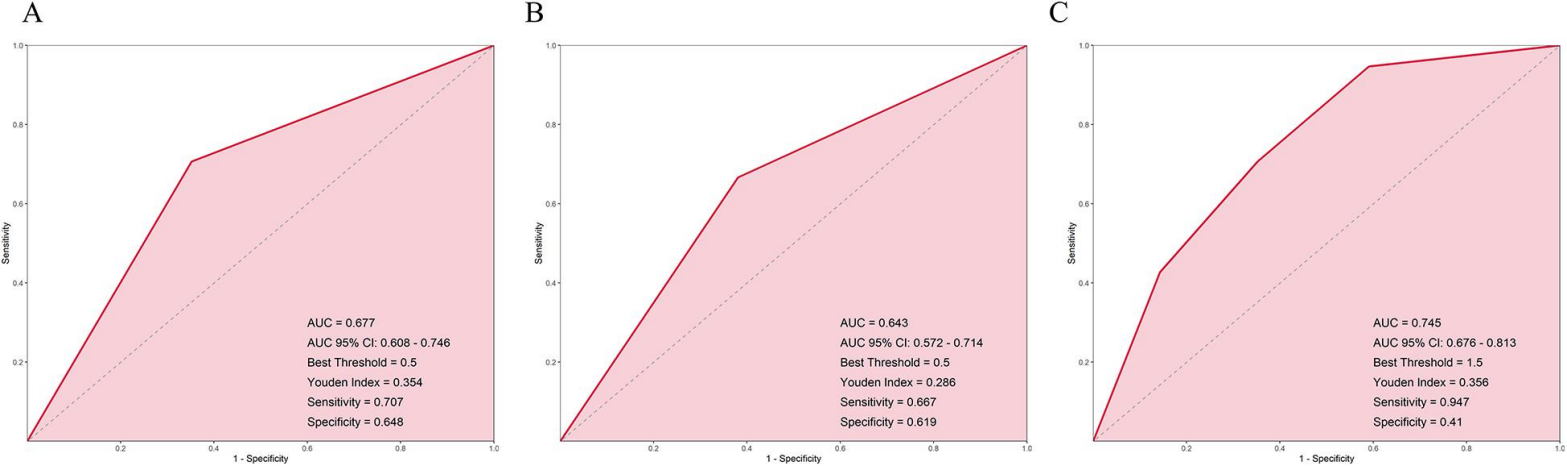
ROC curves for predicting AF recurrence based on **(A)** HRV groups, **(B)** anxiety groups, and **(C)** combined HRV and anxiety interaction groups.

The results showed that the AUC of the HRV model was 0.677, the AUC of the anxiety model was 0.643, and the AUC of the interaction model combining both was 0.745, which was significantly higher than that of the HRV or anxiety models alone (Figures 2A–C). This suggests that combining HRV and anxiety indicators can more accurately predict the risk of recurrence after radiofrequency ablation.

### 3.5 Stratified analysis

**Figure 3:**
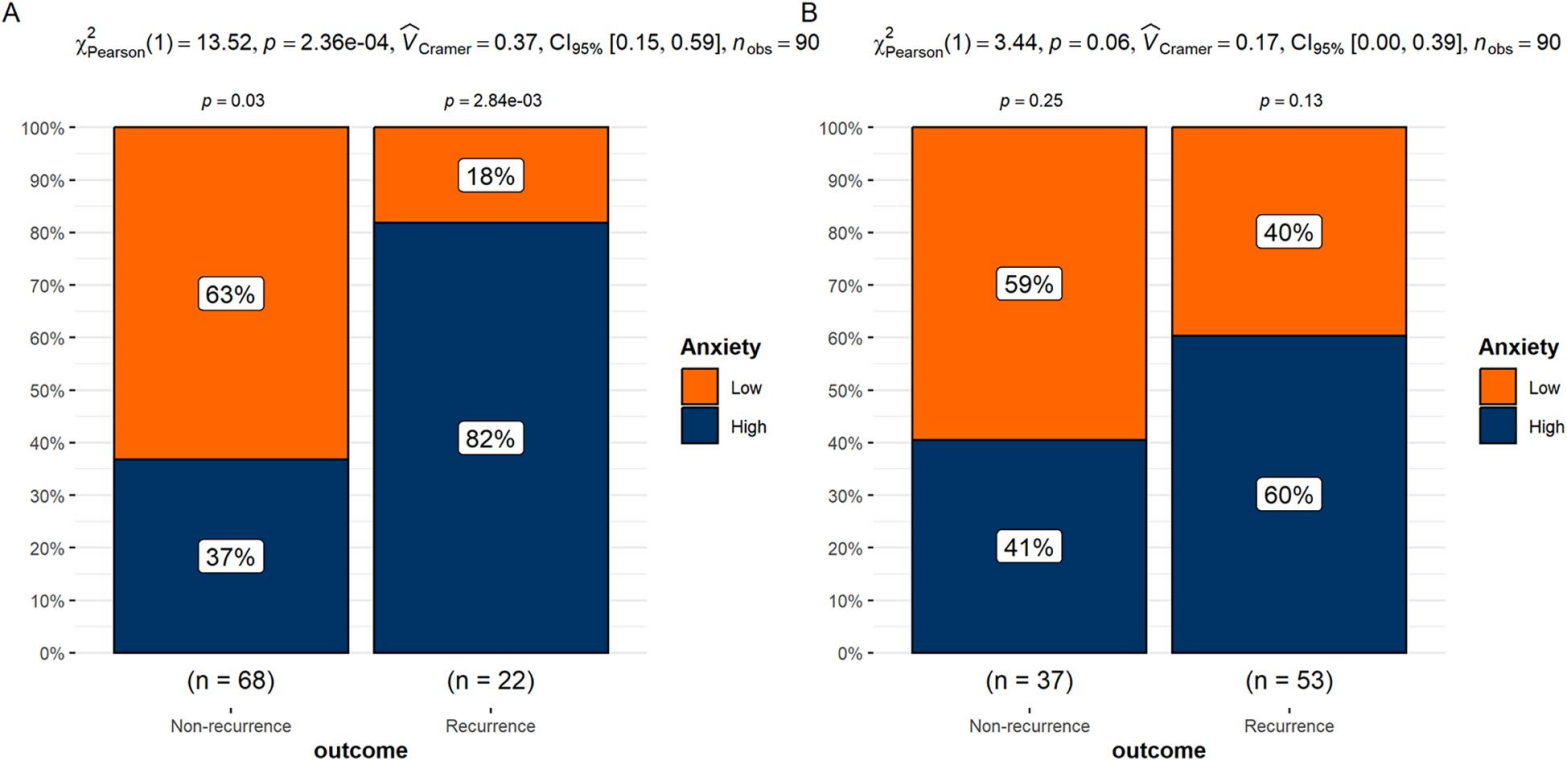
**(A)** Differences in anxiety groups between recurrence and non-recurrence patients in the low HRV group. **(B)** Differences in anxiety groups between recurrence and non-recurrence patients in the high HRV group.

The differences in anxiety levels between the recurrence and non-recurrence groups were analyzed separately within the high and low HRV groups. The results showed that in the low HRV group, the proportion of high-anxiety patients was significantly higher in the recurrence group compared to the non-recurrence group (*P* < 0.05). In the high HRV group, although the recurrence group had a higher proportion of high-anxiety patients than the non-recurrence group, the difference was not statistically significant (*P* > 0.05) (Figures 3A,B).

## 4 Discussion

In the three Cox proportional hazards models we constructed, both anxiety and HRV showed hazard ratios (HR) significantly greater than 1, indicating that higher anxiety levels and lower HRV are both associated with a significantly increased risk of atrial fibrillation (AF) recurrence. This suggests that these two factors hold important predictive value for recurrence. The interaction term also had an HR greater than 1 and was statistically significant (HR = 2.24 in model 1, HR = 1.237 in model 2, and HR = 1.190 in model 3), indicating that anxiety and HRV not only independently affect recurrence risk but may also jointly contribute to an elevated risk through a statistically significant combined effect. In other words, patients with high anxiety and unfavorable HRV levels face a recurrence risk higher than that expected from either factor alone. This supports a multiplicative rather than an additive effect between these variables. The interaction effect remained significant after adjusting for demographic factors (age, sex, BMI) and clinical covariates (heart failure, AF type, disease duration, and cardiac structural parameters) in models 2 and 3, which enhances the credibility and clinical applicability of the findings.

The mechanism underlying the interaction effect of HRV and anxiety on AF recurrence may be as follows: HRV reflects autonomic nervous system function, particularly the balance between sympathetic and parasympathetic activity (18). Reduced HRV typically indicates autonomic dysfunction (19), with increased sympathetic excitation or decreased parasympathetic tone (20). Anxiety activates the stress response system, enhancing sympathetic activity and reducing parasympathetic tone, thereby further lowering HRV (21). This autonomic imbalance promotes myocardial electrophysiological abnormalities and increases susceptibility to arrhythmias, facilitating the occurrence and recurrence of AF (22). Chronic anxiety and psychological stress can also activate the hypothalamic-pituitary-adrenal (HPA) axis (23), elevating inflammatory cytokines such as IL-6 and TNF-α, which may induce early myocardial structural damage. Such damage leads to structural changes in the heart, including atrial enlargement and fibrosis (24), disrupting normal electrical connectivity between cardiomyocytes, forming reentry circuits and electrical instability. Anxiety may further exacerbate these electrophysiological abnormalities through neurotransmitter release, promoting AF recurrence. Low HRV is also linked to elevated inflammation, which may contribute to cardiac electrophysiological disturbances; these factors may mutually reinforce via inflammatory pathways, forming a vicious cycle that increases the risk of AF recurrence. It should be noted, however, that the validity of the LF/HF ratio as a definitive surrogate marker for sympathovagal balance remains controversial. The mechanisms we propose are based on existing literature and remain speculative, as causal pathways are not clearly established and may vary significantly between individuals. Moreover, some studies have suggested that an increase in HRV does not necessarily indicate a favorable prognosis; on the contrary, in some patients, it may reflect enhanced cardiac sympathetic activation, potentially indicating a higher risk of recurrence (25).

We also found that using HRV or anxiety alone to predict AF recurrence had limited predictive ability. However, when combined, the AUC increased to 0.745, indicating relatively improved predictive performance. This highlights the importance of a multidimensional comprehensive assessment. This suggests that patients presenting with both reduced HRV and high anxiety levels may benefit from closer follow-up management. Our findings support the potential role of the interaction between physiological and psychological factors in the mechanism of AF recurrence and provide theoretical support for the design of individualized intervention strategies.

The combined assessment of HRV and anxiety holds broad potential for clinical application. First, this approach can aid in risk stratification, allowing identification of patients at high risk of atrial fibrillation (AF) recurrence after ablation, thereby enabling personalized follow-up plans. For example, in patients with reduced preoperative HRV and high anxiety levels, clinicians may consider increasing the frequency of post-ablation follow-up and implementing more intensive rhythm monitoring. Preoperative improvement of patients' anxiety levels can enhance their treatment adherence, indirectly reducing the risk of postoperative atrial fibrillation recurrence. This study may provide valuable reference information for early identification and psychological counseling interventions for high-risk patients. Due to the long-term imbalance of their autonomic nervous systems, high-risk patients are more likely to benefit from autonomic modulation therapies, including HRV biofeedback training, exercise rehabilitation, and mindfulness or meditation therapies, thereby reducing the risk of postoperative recurrence and improving the scientific rigor and effectiveness of post-ablation management.

In this study, pulmonary vein isolation (PVI) was the primary ablation target for all patients. For selected cases—particularly those with persistent AF or atrial flutter induced by large reentrant circuits confirmed intraoperatively—roof line, mitral isthmus line, or tricuspid isthmus line ablation was selectively added based on intracardiac electrophysiological mapping to enhance conduction block. It is noteworthy that recent randomized controlled trials, such as the EARNEST-PVI study, have not demonstrated a consistent advantage of adjunctive ablation over PVI alone in the general population (26). Moreover, the 2023 ACC/AHA/HRS guidelines for the management of atrial fibrillation (Class of Recommendation 2b, Level of Evidence B-R) also do not support the routine application of such adjunctive ablation strategies (27). Therefore, adjunctive linear ablation should not be routinely performed but rather individualized according to the patient's arrhythmic substrate and electrophysiological characteristics. We also observed that the non-recurrence group had lower preoperative RMSSD levels. This may be because reduced RMSSD typically reflects diminished vagal activity, and low-level vagal stimulation has been shown to significantly reduce the incidence of postoperative atrial fibrillation (POAF) following cardiac surgery (28). However, this finding may also have been influenced by potential confounding factors such as the timing of measurement or sleep quality. Hence, the relationship between RMSSD and the risk of postoperative AF recurrence requires further investigation and validation under conditions where these confounders are well controlled.

This study has certain limitations. First, it is a retrospective study with potential selection bias. Second, data were sourced from a single center with a relatively small sample size. Third, the specific physiological mechanisms underlying the interaction between HRV and anxiety were not experimentally explored. Before changes are made in clinical practice, future studies should consider larger, prospective randomized controlled trials to validate our findings. It should be noted that although the interaction effect remained statistically significant across all three models, the *p*-value was close to the threshold of significance, indicating a marginal effect. As such, this finding is considered a preliminary indication or exploratory observation, and its clinical implications require further validation in studies with larger sample sizes and prospective designs. In this study, the GAD-7 was used to assess anxiety status. This tool is commonly used in primary care settings or as an adjunct in psychiatric or psychological clinics, but it is essentially a screening tool and cannot replace a clinical diagnosis. Moreover, depressive symptoms often coexist with anxiety and may introduce confounding effects. Since this study did not systematically assess depression, future research should include depression scales, such as the PHQ-9, to more comprehensively evaluate the impact of emotional states on HRV and prognosis. Anxiety levels and HRV were assessed only once during the routine preoperative evaluation, without standardized timing or repeated measurements. Future studies may consider multi-time-point assessments.

## 5 Conclusion

This study analyzed the interaction effect of HRV and anxiety on AF recurrence after RFCA by constructing three Cox proportional hazards models. Both reduced HRV and high anxiety levels were identified as independent risk factors for recurrence, with a significant synergistic interaction between them. The ROC curve showed that the combined model of HRV and anxiety yielded an AUC of 0.745, indicating better predictive performance than either factor alone and suggesting some potential value for recurrence risk assessment. Stratified analysis further indicated that, in patients with low HRV, the association between high anxiety and recurrence risk was more pronounced, suggesting the potential relevance of psychological factors in this high-risk subgroup.

## Data Availability

The original contributions presented in the study are included in the article/Supplementary Material, further inquiries can be directed to the corresponding author.
