# Supplementary material for: Prediction of the impact of anxiety on atrial fibrillation recurrence after radiofrequency catheter ablation based on heart rate variability
Source: Front Surg. 2025 Sep 23;12:1653123. doi: 10.3389/fsurg.2025.1653123 (PMC12500613; doi:10.3389/fsurg.2025.1653123)
Supplement: Supplementary file 1 [file Supplementaryfile1.docx]

| **Supplement Table. 1 The interaction effect of GAD-7 and LF/HF on recurrence** | | | | | | |
| --- | --- | --- | --- | --- | --- | --- |
| **Term** | **HR** | **std.error** | **statistic** | **p.value** | **CI-Lower** | **CI-Upper** |
| **GAD-7** | 2.917 | 0.285 | 3.756 | < 0.001 | 1.669 | 5.100 |
| **LF/HF** | 2.072 | 0.337 | 2.162 | 0.031 | 1.070 | 4.011 |
| **Age** | 0.991 | 0.015 | -0.6 | 0.548 | 0.962 | 1.021 |
| **Gender** | 0.97 | 0.265 | -0.114 | 0.909 | 0.577 | 1.631 |
| **BMI** | 0.958 | 0.04 | -1.066 | 0.286 | 0.886 | 1.036 |
| **HF** | 2.375 | 0.319 | 2.716 | 0.007 | 1.271 | 4.438 |
| **Type of Atrial Fibrillation** | 3.276 | 0.25 | 4.751 | < 0.001 | 2.007 | 5.347 |
| **Duration** | 1.314 | 0.089 | 3.077 | 0.002 | 1.104 | 1.564 |
| **EFT** | 1.295 | 0.107 | 2.404 | 0.016 | 1.050 | 1.597 |
| **LAD** | 1.064 | 0.057 | 1.079 | 0.281 | 0.952 | 1.190 |
| **LAVI** | 1.056 | 0.038 | 1.434 | 0.152 | 0.980 | 1.138 |
| **GAD-7*LF/HF** | 2.180 | 0.352 | 2.214 | 0.027 | 1.094 | 4.346 |
